# Acuminosylation of Tyrosol by a Commercial Diglycosidase

**DOI:** 10.3390/ijms24065943

**Published:** 2023-03-21

**Authors:** Peter Haluz, Peter Kis, Matej Cvečko, Mária Mastihubová, Vladimír Mastihuba

**Affiliations:** Institute of Chemistry, Slovak Academy of Sciences, SK-845 38 Bratislava, Slovakia; peter.haluz@savba.sk (P.H.); peter.kis@savba.sk (P.K.); matej.cvecko@savba.sk (M.C.); maria.mastihubova@savba.sk (M.M.)

**Keywords:** diglycosidases, acuminosidase, Aromase H2, transacuminosylation, Osmanthuside H

## Abstract

A commercial glycosidase mixture obtained from *Penicillium multicolor* (Aromase H2) was found to comprise a specific diglycosidase activity, β-acuminosidase, alongside undetectable levels of β-apiosidase. The enzyme was tested in the transglycosylation of tyrosol using 4-nitrophenyl β-acuminoside as the diglycosyl donor. The reaction was not chemoselective, providing a mixture of Osmanthuside H and its counterpart regioisomer 4-(2-hydroxyethyl)phenyl β-acuminoside in 58% yield. Aromase H2 is therefore the first commercial β-acuminosidase which is also able to glycosylate phenolic acceptors.

## 1. Introduction

**Figure 1:**
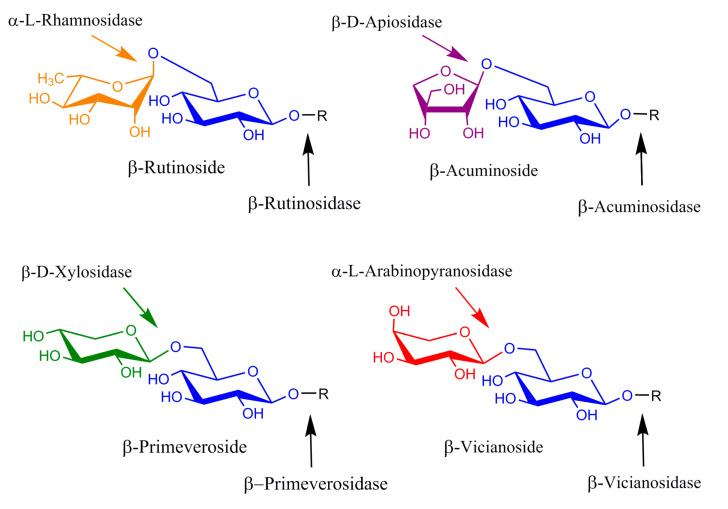
Diglycosidases and exoglycosidases: substrates and cleavage sites.

Diglycosidases represent an interesting group of β-endoglycosidases catalyzing the one-step hydrolysis of diglycosides. The group includes acuminosidases (6-O-β-D-apiosyl-β-D-glucosidases), primeverosidases (6-O-β-D-xylopyranosyl-β-D-glucosidases), vicianosidases (6-O-α-L-arabinopyranosyl-β-D-glucosidases) and rutinosidases/hesperidinases (6-O-α-L-rhamnopyranosyl-β-D-glucosidases). Although originally discovered in plants, where they play roles in plant defense systems through the release of toxic, deteriorating or semiotic substances, diglycosidases were also found to be produced by various types of micro-organisms. Their common feature is that, contrary to exoglycosidases, they do not hydrolyze oligosaccharides and diglycosides by the stepwise release of monosaccharides; instead, they specifically hydrolyze the β-glycosidic bond between the disaccharide and the aglycone (Figure 1). Several diglycosidases were sequenced and assigned to the families of glycoside hydrolases GH1 (retaining plant diglycosidases [1,2,3]), GH3 (promiscuous retaining rutinosidase from *Acremonium* sp. [4]), GH5-23 (retaining microbial primeverosidases [5], rutinosidases [6] and hesperidinases [7] related to exo-1,3-β-glucanases) and GH 55 (inverting rutinosidase from *Actinoplanes missouriensis* [8]). The industrial potential of diglycosidases is in the food and beverage industries for the modulation of flavor [9,10,11]. The enzymes may, however, find a use in biocatalysis for the production of tailor-made structured glycosides and oligosaccharides. Rutinosidases and hesperidinases, the enzymes releasing glycosidically bound rutinose, are the most commonly studied group of diglycosidases due to the widespread availability and the low price of their natural substrates, rutin and hesperidin. Being of either plant or microbial origin, they find use, for example, in the debittering and clarification of citrus juices or wine aroma development, and they have been successfully used in the rutinosylation of several aliphatic, terpenic or phenolic substances [12]. Primeverosidases are an important catalyst for the development of the flavor of green tea by the release of terpenic alcohols from their glycosidic precursors, and they may also find use in the modulation of the aroma of wine.

Acuminosidases are the least commonly explored diglycosidases for several reasons: there is only one natural acuminoside, furcatin (p-allylphenyl-β-acuminoside), which has been isolated in reasonable amounts from the leaves of the Japanese honeysuckle bush *Viburnum furcatum* [13]; the chemical synthesis of probes for acuminosidase is challenging, and their enzymatic synthesis would require high amounts of free apiose due to the inverting mechanism of commercially available apiosidases [14]; and with the exception of wine and tea aroma development from acuminoside precursors, there is no present proof of the effective application for acuminosidases. An acuminoside motif, however, frequently occurs in secondary metabolites of various medicinal plants [15,16,17,18,19,20]. The enzymes may therefore find application as biocatalysts in the preparation of the known pharmacoactive substances or their new analogues. There are several known sources of diglycosidases comprising acuminoside hydrolyzing activity, which are most commonly of plant origin. In 1975, Hösel and Barz [21] reported the release of disaccharide from apioglucosylated Biochanin A by chickpea β-glucosidases. Primeverosidase from tea leaves hydrolyzes, alongside primeverosides, also other diglycosidic aroma precursors, including acuminosides [22]. Grape skins were reported to contain β-endoglycosidases with wide-ranging substrate specificity and also hydrolyze grape monoterpenyl acuminosides [23]. A glycosidase extracted from *Dalbergia nigrescens* was found to hydrolyze acuminosides of dalnigrein and dalpatein [24,25]. Yet the most promising and the most widely studied acuminosidase is furcatin hydrolase from the leaves of *V. furcatum*, which together with furcatin is a component of the plant defense system responsible for the fast release of p-allylphenol during attacks by herbivores [26]. The enzyme was first described as early as 1961 [27] and tested through transacuminosylations of short aliphatic alcohols to obtain simple acuminosides. Furcatin served as the donor of an acuminosyl moiety. Furcatin hydrolase has a 64% sequence identity with the tea leaf β-primeverosidase [2,26] and also hydrolyzes, to some extent, phenolic β-primeverosides, β-glucopyranosides and vicianin. The enzyme has been cloned and expressed in *Escherichia coli*; its wider application in biocatalysis is, however, hampered by the prices of the substrate furcatin, which are prohibitive of reactions on a synthetic scale. An in-house isolation of furcatin is complicated by limited access to the plant material outside its country of origin.

At the start of the millennium, Tsuruhami and his coworkers studied a β-primeverosidase-like enzyme produced by *Penicillium multicolor* [5,28]. The purified enzyme displayed furcatin hydrolyzing activity on the level of about 1% of that of β-primeverosidase and was used in the synthesis of 2-phenylethyl β-primeveroside. Recently, the enzyme company Amano released a preparation of glycosidases from *Penicillium multicolor* comprising β-primeverosidase (Aromase H2) for the enhancement of the aroma of wine, fruit juices, tea and botanicals through the hydrolysis of diglycoside aroma precursors. This is probably the first commercial diglycosidase preparation on the market that is available in kilogram amounts, thus opening up opportunities for biocatalytic applications.

As a continuation of our research on the synthetic use of diglycosidases and apiosidase-related glycosidases, we studied the potential of this promising biocatalyst. The purpose of this short communication is to demonstrate that Aromase H2 displays, in addition to β-primeverosidase at least one more diglycosidase activity—acuminosidase—in levels which are practical for its use in syntheses without any purification and to demonstrate the application of Aromase H2 in the acuminosylation of tyrosol. The chemoselectivity of the reaction is also briefly discussed.

## 2. Results and Discussion

Since diglycosidases compete for their substrates with mono-glycosidases (exoglycosidases), it is important to distinguish these two types of activities in order to verify whether the hydrolysis of diglycoside is catalyzed by the diglycosidase. Moreover, exoglycosidases can affect the synthesis of diglycosides by the secondary hydrolysis of the product or may glycosylate the aglycone by the monosaccharide from the nonreducing end of the substrate. As seen in Table 1, Aromase H2 is a cocktail of various mono- and diglycosidase activities. It displays rutinosidase, primeverosidase and vicianosidase activities on the levels of 20.5, 0.016 and 0.5 kU/g. These activities should, however, be considered as apparent, since the enzyme preparation also comprises α-L-rhamnosidase, β-D-xylosidase and α-L-arabinopyranosidase activities as high as 44, 1.6 and 0.9 kU/g, respectively. This fact (together with the activity of β-D-glucosidase, ca. 3.7 kU/g) suggests that Aromase H2 cannot be directly used in transrutinosylations, transprimeverosidations or transvicianosidations without the removal of monoglycosidases. On the other hand, Aromase H2 comprises high levels of acuminosidase (ca. 1 kU/g) along with undetectable β-apiosidase activity. The acuminosidase is therefore a real activity without the contribution of monoglycosidase, and the enzyme can be used in the synthesis of acuminosides with a low risk of side reactions. The absence of the apiosidase is in agreement with our finding that, with the exception of a rather curious apiosidase from *Bacteroides thetaiotaomicron* [29], outside of the plant kingdom this enzyme was found to be produced only by aspergilli [30].

**Figure 2:**
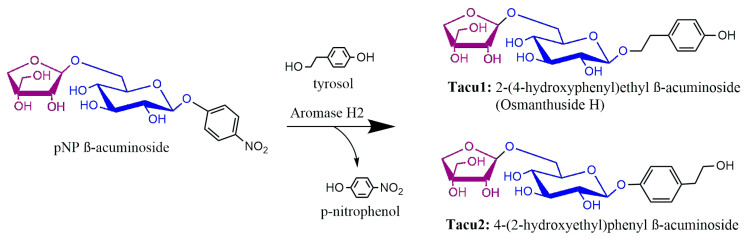
Acuminosylation of tyrosol by Aromase H2.

Driven by our continual interest in the preparation of oligosaccharides glycosylated by phenylethanoid alcohols, we decided to perform the transacuminosylation of tyrosol starting with 4-nitrophenyl β-acuminoside. This serves as both a chromogenic probe for assaying acuminosidase activity and the activated substrate for transglycosylations (Figure 2). The reaction proceeded smoothly under conditions for hydrolysis recommended by the producer, providing, in a yield of ca. 58%, a mixture of two diglycosides: **Tacu1**, i.e., Osmanthuside H, and its phenol-acuminosylated regioisomer, **Tacu2**, in an approximate ratio of 1:0.9. We were, however, unable to separate the products each from other.

The molecular formula was established as C_19_H_28_O_11_ by HRMS (ESI). The ^1^H- and ^13^C NMR spectra revealed the presence of β-D-apiofuranose moieties linked through a 1→6 glycosidic bond to glucopyranose. The β-anomeric configuration for the glucopyranoses was determined from ^3^J_H1,H2_ coupling constants values (7.8 and 7.7 Hz). The stereochemistry of the anomeric protons of glucose and apiose were also determined as β from the chemical shifts of C-1 and C-1′ (104.4 and 111.0 for **Tacu1**, 102.6 and 110.9 for **Tacu2**). The aglycone of **Tacu1** showed A_2_B_2_-type aromatic protons (δ 7.07 (d, *J* = 8.5 Hz, 2H, Ph) and 6.70 (d, *J* = 8.6 Hz, 2H, Ph)), and the two methylenes of 4-hydroxyphenylethanol part CH_2_β displayed one signal (δ 2.84 (ddd, *J* = 8.7, 7.0, 2.2 Hz, 2H, CH_2_β)), while CH_2_α showed two separated proton signals (δ 4.03–3.94 (m, 1H, OCH_2_αa) and 3.73–3.67 (m, 1H, CH_2_αb)). The ^1^H NMR spectrum of **Tacu2** displays a set of proton signals due to the aromatic AA’BB’ spin system indicative of the 1,4-disubstituted phenyl ring at δ 7.16 (d, *J* = 8.6 Hz, 2H, Ph) and 7.03 (d, *J* = 8.5 Hz, 2H, Ph), as well as a benzylic methylene at δ 2.77 (t, *J* = 7.1, Hz, 2H, CH_2_β) and a hydroxymethyl at 3.72 (t, 2H, *J* = 7.1 Hz, OCH_2_α), indicating the presence of a 2-(4-hydroxyphenyl)ethanol moiety and the glucosylation of the phenolic OH. 

The acuminosylation of tyrosol is therefore not chemoselective, and to obtain a single regioisomer, the selective protection of tyrosol is necessary. Acetylation, as a protection method, is ineffective, since Aromase H2 comprises high levels of acetyl esterase activity, hydrolyzing the tyrosol acetate. On the other hand, silylation or benzylation diminish the solubility of the protected tyrosol in the reaction media. 

Low chemoselectivity in the glycosylation of tyrosol seems to be more typical of microbial glycosidases. Recently, we used the dried flower buds of *Sophora japonica* for the rutinosylation of tyrosol. The reaction proceeded in high yields with an absolute chemoselectivity toward the primary hydroxyl [31]. On the other hand, Bassanini et al. [32] realized the same reaction with rutinosidase from *Aspergillus niger* and observed the formation of a mixture of product regioisomers with a 25% content of the product with rutinosylated phenolic hydroxyl. Although the low chemoselectivity of acuminosidase from *Penicillum multicolor* is not advantageous for the glycosylation of molecules bearing several hydroxyl groups of different chemical natures, the ability to glycosylate a phenolic moiety is not very common and makes this enzyme a very valuable biocatalyst.

**Figure 3:**
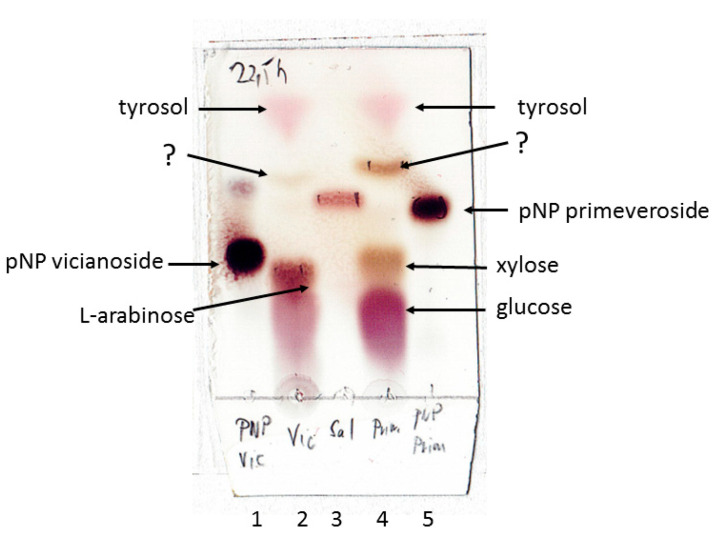
Products of transglycosylations of tyrosol from pNP vicianoside and pNP primeveroside catalyzed by Aromase H2. Line 1: standard of pNP vicianoside. Line 2: reaction of pNP vicianoside with tyrosol. Line 3: standard of salidroside. Line 4: reaction of pNP primeveroside with tyrosol. Line 5: standard of pNP primeveroside.

Although we found several apparent diglycosidase activities in Aromase H2, we cannot assume their real levels without their isolation due to high contents of disturbing exoglycosidases. Moreover, it is not clear whether Aromase H2 contains only one diglycosidase with a wide-ranging substrate specificity or whether several diglycosidases occur. The results of the vicianosylation and primeverosylation of tyrosol on an analytical scale with non-purified Aromase H2 suggest the formation of tyrosol β-D-xylopyranoside and traces of tyrosol L-arabinoside visible upon the TLC analysis of reaction mixtures (Figure 3) as spots, with retention factors situated slightly above the spot of salidroside (tyrosol β-D-glucopyranoside). Non-purified Aromase H2 cannot, therefore, be used for diglycosylations other than acuminosylation.

## 3. Materials and Methods

4-Nitrophenyl α-D-glucopyranoside, 4-nitrophenyl β-D-glucopyranoside, 4-nitrophenyl α-D-galactopyranoside, 2-nitrophenyl β-D-galactopyranoside, 4-nitrophenyl α-L-arabinopyranoside, 4-nitrophenyl α-L-arabinofuranoside, 4-nitrophenyl α-L-rhamnopyranoside, 4-nitrophenyl β-D-mannopyranoside and 4-nitrophenyl β-D-xylopyranoside were products from Biosynth (Bratislava, Slovakia). 4-Nitrophenyl acetate was purchased from Merck Slovakia (Bratislava, Slovakia). Tyrosol (97%) was purchased from Maybridge (Loughborough, Leicestershire, UK). 4-Nitrophenyl β-D-apiofuranoside, 4-nitrophenyl rutinoside, 4-nitrophenyl acuminoside, 4-nitrophenyl primeveroside and 4-nitrophenyl vicianoside were products of the Institute of Chemistry, SAS (Bratislava, Slovakia). Salidroside was kindly gifted by Dr. Elena Karnišová Potocká, Institute of Chemistry, SAS (Bratislava, Slovakia). Aromase H2 was generously gifted by Dr. Fumiaki Ito, Amano Enzyme Europe Limited (Oxfordshire, UK). Silica gel 60 for flash chromatography was obtained from Fluka (Buchs, Switzerland). TLC was performed on alumina plates with Silica gel 60 F254 from Merck KGaA (Darmstadt, Germany).

Flash chromatography was performed on Isolera One from Biotage (Uppsala, Sweden), with UV detection. The structures of the products were determined by a combination of ^1^H and ^13^C NMR spectroscopy as well as two-dimensional homonuclear and heteronuclear techniques (COSY, HSQC) and recorded on a 400 MHz Bruker AVANCE III HD equipped with a Prodigy CryoProbe. Chemical shifts are reported in ppm (δ) and are referenced to the internal CD_3_OD (δ 3.31, for ^1^H, and δ 49.00, for ^13^C). Vicinal couplings are reported in hertz (Hz). High-resolution mass determination was performed using an Orbitrap Velos Pro Thermo Scientific mass analyzer (ion source HESI, capillary temperature 350 °C, source heater temperature 300 °C, mass range 80–600 *m*/*z*, full scan, positive polarity, resolution 120,000.)

The assay of the glycosidase activities was as follows: 50 µL of properly diluted enzyme was mixed with 0.9 mL of 0.1 M acetate buffer pH 5 and 50 µL of 0.1 M (resp. 0.02 M for diglycosides) substrate and incubated at 50 °C and 500 rpm. Aliquots were withdrawn after 5 and 10 min and mixed with 5 volumes of a saturated solution of borax and then filtered, and the formation of free nitrophenol was measured at 410 nm. One glycosidase unit corresponds to the amount of enzyme catalyzing the hydrolysis of 1 µmol of substrate in one minute. The activity was calculated from data obtained after 5 min of reaction; measurements taken after 10 min served as a control of the linearity of the reaction.

Acetyl esterase was measured using 4-nitrophenyl acetate according to the method used in our previous work [31].

For the preparative acuminosylation of tyrosol, the preparation of tyrosyl acuminoside was performed with 0.05 M pNP acuminoside as a saccharide donor in a volume of 10 mL. The final concentration of tyrosol was 0.2 M, and the reaction started with the addition of 0.5 mL of diluted enzyme (10 mg/mL). The reaction was incubated at 50 °C and stopped after 5 h by boiling for 10 min in a water bath and passing through a 0.22 µm filter. The filtrate was applied to a column of Diaion HP-20 equilibrated and eluted with water to wash away the free saccharides. The product and free tyrosol were then eluted with 10% ethanol. Fractions containing the product were collected, concentrated in vacuo, applied to a silica gel column and eluted with a gradient of methanol in chloroform. The structures of the isolated products were confirmed by NMR and MS spectroscopy.

Mixture Tacu1/Tacu2 = 1:0.9; waxy solid; HRMS (ESI): *m*/*z* calcd for C_19_H_28_O_11_Na ([M + Na]^+^) 455.15238, found 455.15213; calcd for C_19_H_28_O_11_K ([M + K]^+^) 471.12632, found 471.12578.

The transglycosylations on an analytical scale were performed under the same conditions as the acuminosylation with the following changes: the substrates were pNP primeveroside and pNP vicianoside, and the volume of the reaction was 1 mL in Eppendorf tubes shaken at 400 rpm. The reaction was followed by TLC, using a mixture of chloroform/methanol/water 6.0:3.5:0.5.

### 3.1. NMR Data of Prepared Acuminosides

#### 3.1.1. 2-(4-Hydroxyphenyl)ethyl 6-O-β-D-apiofuranosyl-β-D-glucopyranoside (2-(4-hydroxyphenyl)ethyl acuminoside, **Tacu1**)

^1^H NMR (400 MHz, CD_3_OD) δ 7.07 (d, *J* = 8.5 Hz, 2H, Ph), 6.70 (d, *J* = 8.6 Hz, 2H, Ph), 4.98 (d, *J* = 2.4 Hz, 1H, H-1′), 4.28 (d, *J* = 7.8 Hz, 1H, H-1), 4.03–3.96 (m, 1H, H-6a), 4.03 -3.94 (m, 1H, OCH_2_αa), 3.61 (dd, *J* = 11.1, 6.2 Hz, 1H, H-6b), 3.97 (d, *J* = 9.6 Hz, 1H, H-4′a), 3.92 (d, *J* = 2.5 Hz, 1H, H-2′), 3.76 (d, *J* = 9.7 Hz, 1H, H-4′b), 3.73–3.67 (m, 1H, CH_2_αb), 3.58 (s, 2H, H-5′ab), 3.42–3.36 (m, 1H, H-5), 3.34 (t, *J* = 8.8 Hz, 1H, H-3), 3.27 (dd, *J* = 9.6, 8.8 Hz, 1H, H-4), 3.17 (dd, *J* = 9.0, 7.9 Hz, 1H, H-2), 2.84 (ddd, *J* = 8.7, 7.0, 2.2 Hz, 2H, CH_2_β).

^13^C NMR (101 MHz, CD_3_OD) δ 156.8 (C-Ph), 130.9 (2xCH-Ph), 130.7 (C-Ph), 116.1 (2xCH-Ph), 111.0 (C-1′), 104.4 (C-1), 80.6 (C-3′), 78.1 (C-2′), 78.0 (C-3), 76.9 (C-5), 75.1 (C-2), 74.9 (C-4′), 72.2 (CH_2_α), 71.7 (C-4), 68.8 (C-6), 65.6 (C-5′), 36.4 (CH_2_β).

#### 3.1.2. 4-(2-Hydroxyethyl)phenyl 6-O-β-D-apiofuranosyl-β-D-glucopyranoside (4-(2-hydroxyethyl)phenyl acuminoside, **Tacu2**)

^1^H NMR (400 MHz, CD_3_OD) δ 7.16 (d, *J* = 8.6 Hz, 2H, Ph), 7.03 (d, *J* = 8.5 Hz, 2H, Ph), 5.01 (d, *J* = 2.5 Hz, 1H, H-1′), 4.82 (d, *J* = 7.7 Hz, 1H, H-1), 4.03–3.96 (m, 1H, H-6a), 3.72 (t, 2H, *J* = 7.1 Hz, OCH_2_α), 3.60 (dd, *J* = 11.1, 6.1 Hz, 1H, H-6b), 3.96 (d, *J* = 9.8 Hz, 1H, H-4′a), 3.91 (d, *J* = 2.5 Hz, 1H, H-2′), 3.77 (d, *J* = 9.7 Hz, 1H, H-4′b), 3.60–3.54 (m, 1H, H-5), 3.59 (s, 2H, H-5′ab), 3.45 (t, *J* = 8.2 Hz, 1H, H-3), 3.43 (dd, *J* = 8.3, 7.1 Hz, 1H, H-2), 3.34 (t, 8.8 Hz, 1H, H-4), 2.77 (t, *J* = 7.1, Hz, 2H, CH_2_β).

^13^C NMR (101 MHz, CD_3_OD) δ 157.6 (C-Ph), 130.9 (2xCH-Ph), 134.3 (C-Ph), 117.9 (2xCH-Ph), 110.9 (C-1′), 102.6 (C-1), 80.6 (C-3′), 78.2 (C-2′), 78.0 (C-3), 77.0 (C-5), 74.9 (C-2), 74.9 (C-4′), 71.7 (C-4), 68.7 (C-6), 65.6 (C-5′), 64.4 (CH_2_α), 39.4 (CH_2_β).

## 4. Conclusions

The commercial glycosidase cocktail Aromase H2, derived from *Penicillium multicolor*, comprises a high level of β-acuminosidase with undetected apiosidase activity. It can therefore be used directly from the bottle in the β-acuminosylation of tyrosol. The reaction provides an approximately equimolar mixture of naturally occurring Osmanthuside H and its regioisomer: the phenolic β-acuminoside of tyrosol. According to our knowledge, Aromase H2 is the first commercial source of β-acuminosidase activity catalyzing transacuminosylation. Its ability to glycosylate phenols makes it a prospective biocatalyst with a reactivity distinct from that of common glycosidases that widens the scale of enzymatically preparable glycophenolics. Synthetic applications of its diglycosidase activities, with the exception of acuminosidase, however, require purification steps to remove the intriguing exoglycosidases.

## Figures and Tables

**Table 1:** Glycosidase activities in Aromase H2.

Activity	U/g	Substrate
α-D-Glucosidase	506 ± 72	pNP α-D-glucopyranoside
β-D-Glucosidase	3657 ± 275	pNP β-D-glucopyranoside
α-D-Galactosidase	2024 ± 173	pNP α-D-galactopyranoside
β-D-Galactosidase	2920 ± 376	oNP β-D-galactopyranoside
α-L-Arabinofuranosidase	25,200 ± 3137	pNP α-L-arabinofuranoside
α-L-Arabinopyranosidase	882 ± 43	pNP α-L-arabinopyranoside
α-L-Rhamnosidase	43,995 ± 3658	pNP α-L-rhamnopyranoside
β-D-Mannosidase	n.d.	pNP β-D-mannopyranoside
β-D-Apiosidase	n.d.	pNP β-D-apiofuranoside
β-D-Xylosidase	1634 ± 101	pNP β-D-xylopyranoside
β-Rutinosidase	20,472 ± 2227	pNP β-rutinoside
β-Acuminosidase	979 ± 127	pNP β-acuminoside
β-Vicianosidase	466 ± 18	pNP β-vicianoside
β-Primeverosidase	16 ± 0.2	pNP β-primeveroside

## Data Availability

Data are contained within the article.
